# Enzyme Architecture: Activation of Phosphite Dehydrogenase-Catalyzed Hydride Transfer by NAD^+^ Cofactor Fragments

**DOI:** 10.1021/acs.biochem.5c00561

**Published:** 2025-11-11

**Authors:** Rania Hegazy, John P. Richard

**Affiliations:** Department of Chemistry, 12292 University at Buffalo, SUNY, Buffalo, New York 14260-3000, United States

## Abstract

We report the results of experiments to test the hypothesis that binding energy from the adenosine diphosphate (ADP) fragment of the NAD^+^ cofactor is utilized to drive a protein conformational change that activates phosphite dehydrogenase (PTDH) for catalysis of hydride transfer from phosphite to NAD^+^. The ADP fragment of the NAD^+^ cofactor provides >8.5 kcal/mol stabilization of the transition state for PTDH-catalyzed hydride transfer. The ADP and AMP fragments of NAD^+^ activate PTDH for catalysis of hydride transfer from phosphite to nicotinamide riboside (NR). At a 1.0 M standard state these activators stabilize the hydride transfer transition state by 5.1 (ADP) and 2.7 (AMP) kcal/mol, so the activation is due to protein interactions with both the α- and β-ADP phosphates. There is no detectable stabilization of the transition state for PTDH-catalyzed hydride transfer to NR by the adenosine fragment of NAD^+^. Activation is proposed to result from stabilization of the closed form of PTDH by a cation–anion pair with the K76 side chain that bridges the α- and β-phosphates of NAD^+^. By comparison, the activation of formate dehydrogenase- and glycerol phosphate dehydrogenase-catalyzed hydride transfer by ADP is from enzyme interactions with the α-phosphate of ADP, with little or no contribution from the β-phosphate. These results show a diversity in the evolution of enzyme-activating conformational changes for dehydrogenase-catalyzed hydride transfer reactions.

## Graphical abstract

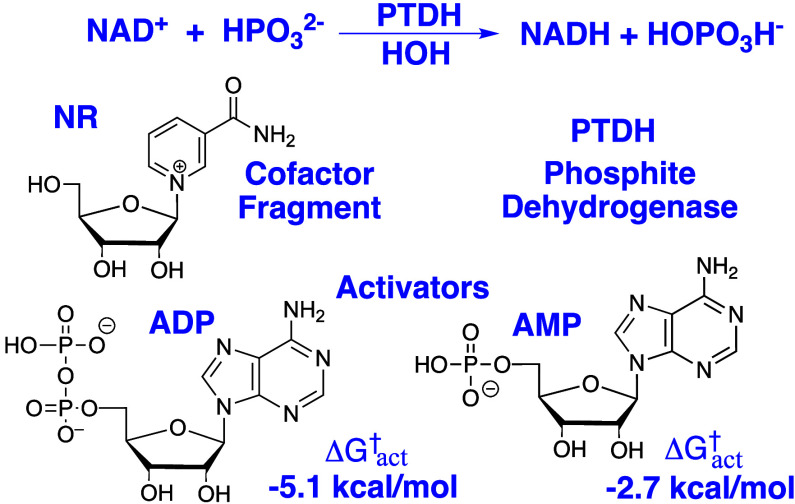

## Introduction

Enzymes operate by utilizing protein–substrate interactions for stabilization of catalyzed reaction transition states.^1^ The total transition state stabilization for many enzymes is so large that its full expression at the Michaelis complex would result in tight and effectively irreversible ligand binding.^2,3^ In many cases this is avoided by instead using binding energy from nonreacting substrate fragments to drive thermodynamically unfavorable protein conformational changes to form catalytically active enzymes.^4–6^ The substrates for these enzymes have been split into two smaller fragments, where the reactive piece undergoes a slow enzyme-catalyzed reaction and the nonreacting piece provides binding energy to drive a protein conformational change that activates the enzyme for catalysis at the reacting piece.^5,6^ Protein conformational changes driven by interactions with the nonreacting phosphodianion fragment of alkyl phosphate substrates give rise to phosphite dianion activation of enzymes for catalysis of proton transfer (triosephosphate isomerase (TIM) and glucose-6-phosphate isomerase),^7–11^ hydride transfer (glycerol phosphate dehydrogenase (GPDH) and glucose-6-phosphate dehydrogenase),^9,12–15^ and decarboxylation (orotidine 5′-monophosphate decarboxylase (OMPDC) and 6-phosphogluconate decarboxylase) reactions of the corresponding phosphodianion truncated substrates.^9,16,17^

Enzyme cofactors consist of compact reactive functional groups attached to nonreacting accessory handles that do not participate directly in the reaction chemistry but provide binding energy to drive protein conformational changes.^18–24^ Succinyl CoA/acetoacetate CoA transferase (SCOT) catalyzes transfer of -SCoA between two carboxylic acids. The Michaelis complexes for SCOT are only modestly stabilized by interactions with the CoA substrate fragment. William Jencks and co-workers proposed that this fragment instead provides the large binding energy required to stabilize the transition state for thiol transfer; and, his laboratory provided extensive experimental evidence to support this proposal.^25–27^ A cofactor driven protein conformational change has been documented for SCOT,^28^ but there have been no reports of experiments to probe its role in activation of SCOT for catalysis of thiol transfer.

We are examining enzyme-catalyzed reactions of NAD^+^ and FAD cofactors in order to extend the earlier studies on SCOT-catalyzed reactions of coenzyme A esters.^29–31^ NAD^+^ consists of the nicotinamide ring hydride acceptor and an adenosine 5′-diphosphate ribose handle (ADPR, Scheme 1) that provides binding energy for transition state stabilization. Formate dehydrogenase (FDH) and GPDH use the binding energy of the ADP fragment of the NAD^+^ cofactor to drive protein conformational changes.^12,20^ Both enzymes catalyze slow hydride transfer from substrate to the truncated nicotinamide riboside (NR) cofactor (Scheme 2). Both enzymes are activated for catalysis of hydride transfer to NR by added adenosine 5′-phosphate (AMP) and adenosine 5′-diphosphate (ADP) fragments of the NAD^+^ cofactor (Scheme 1).^30,31^ We now extend these results to enzyme-catalyzed reactions of NAD^+^ cofactor pieces in the oxidative hydrolysis reaction catalyzed by phosphite dehydrogenase (PTDH, Scheme 2).^32,33^ This enzyme was targeted for the following reasons.

**Scheme 1:**
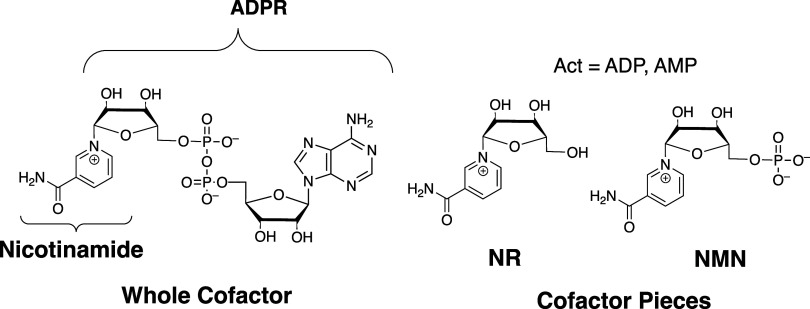
NAD^+^ Cofactor and Representative Cofactor Fragments

**Scheme 2:**
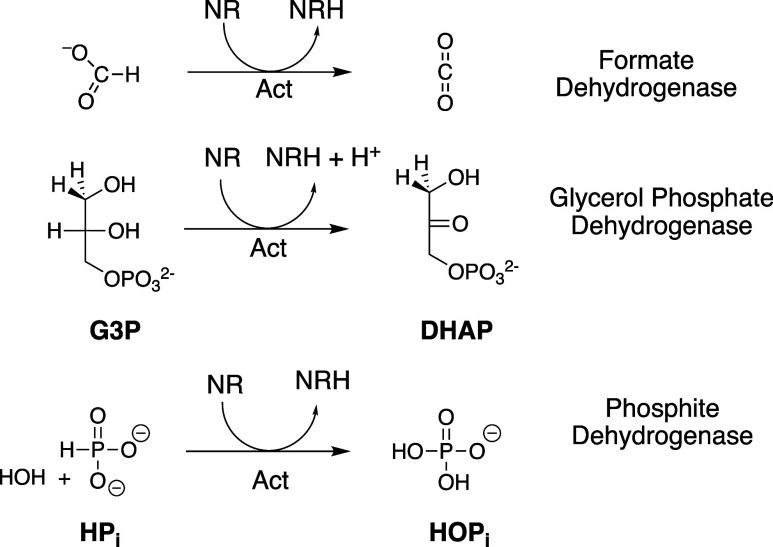
Enzyme-Catalyzed Reactions of Truncated Cofactor NR that are Activated by Cofactor Pieces

(1) The formate monoanion hydride donor for FDH provides minimal binding energy for transition state stabilization, and this creates a strong imperative to utilize binding interactions with the nonreacting fragments of the NAD^+^ cofactor for this purpose.^31^ By comparison, the phosphite dianion hydride donor for PTDH provides up to 12 kcal/mol binding energy for transition state stabilization.^5,34^ However, the primary and secondary ^18^O isotope effects on PTDH-catalyzed hydride transfer to NAD^+^ provide evidence for an associative reaction mechanism that is favored for nucleophilic addition of water to phosphite monoanion compared to the dianion.^35,36^ Results from computational studies provide additional evidence that enzyme-bound phosphite dianion is protonated prior to PTDH-catalyzed nucleophile addition of water to the substrate monoanion.^37,38^ These experimental and computational results predict that FDH and PTDH show similar imperatives for utilization of binding interactions with the ADP-ribose fragment in stabilization of the hydride-transfer transition state. This favors similar fragment activation of enzyme-catalyzed hydride transfer to NR.
(2) The sequence of PTDH has been found to be *between 26 and 34%* identical to the sequences of the top 50 matches to other members of the NAD^+^-dependent D-2-hydroxyacid dehydrogenase family.^39^ This supports a common hydride transfer reaction mechanism for all family members. If so, then the observation of AMP and/or ADP activation of PTDH-catalyzed hydride transfer from HPO_3_^2–^ to the NR cofactor piece (Scheme 2) would provide the testable hypothesis that other members of this family utilize the binding energy of the ADP cofactor-fragment of NAD^+^ for stabilization of the hydride transfer transition state.

We report kinetic parameters for activation of PTDH-catalyzed hydride transfer from phosphite to NR by fragments of the whole NAD^+^ cofactor. The total activation by the ADP fragment is (2–4)-kcal/mol smaller than observed for catalysis by FDH and GPDH. The activation of FDH and GPDH by the ADP cofactor fragment includes a large contribution from binding interactions between the protein catalyst and the ADP α–phosphate but a minimal contribution from interactions with the β-phosphate.^30,31^ By comparison, ADP activation of PTDH-catalyzed hydride transfer to NR involves contributions from protein interactions with both the α–phosphate and β-phosphate of ADP. The results show that the evolution of enzymes that catalyze hydride transfer to NAD^+^ has given rise to variety in the protein–ligand interactions that activate these dehydrogenases for catalysis.

## Experimental Section

### Materials

Water was from a Milli-Q academic purification system. Bovine serum albumin (BSA) fraction V was from Roche Diagnostics. The following reagent grade chemicals were obtained from Sigma-Aldrich: triethanolamine hydrochloride (TEA·HCl), sodium phosphite dibasic pentahydrate, adenosine (Ado), adenosine 5′-monophosphate (AMP, monosodium salt), adenosine 5′-diphosphate (ADP, monosodium salt), and β-nicotinamide adenine dinucleotide sodium salt (NAD^+^). Nicotinamide riboside hydrochloride (NR) was purchased from Selleckchem. Solution pH was determined at 25 °C using an Orion Model 720A pH meter equipped with a Radiometer pHC4006–9 combination electrode that was standardized at pH 4.00, 7.00, and 10.00 at 25 °C. Human adenylate kinase was expressed and purified by a published procedure.^40^ Rabbit muscle pyruvate kinase and porcine heart lactate dehydrogenase were purchased from Calzyme. Formate dehydrogenase from *Candida boidinii* (*Cb*FDH) was purchased from Neogen (Megazyme). Wild-type glycerol 3-phosphate dehydrogenase from human liver (*hl*GPDH) was expressed and purified by a published procedure.^34^ Stock solutions of AMP, ADP, and adenosine were adjusted to pH 7.5 prior to use. The concentration of AMP and ADP was determined enzymatically by published procedures.^30^ The concentration of adenosine was determined from the absorbance at 259 nm using an extinction coefficient of 15,400 M^–1^ cm^–1^.^41^ Stock solutions of phosphite dianion were prepared by following a published procedure and were stored at −20 °C.^42^

The plasmid 17X-PTDH_pET15b containing the gene for 17X-PTDH from *Pseudomonas stutzeri* (17X-*Ps*PTDH) was a generous gift from Professor Graeme Howe at Queens University. This plasmid was prepared previously by introducing the following mutations into the sequence for the parent enzyme: D13E, M26I, V71I, E130K, Q132R, Q137R, I150F, Q215L, R275Q, L276Q, I313L, V315A, A319E, A325V, E332N, C336D; and E175A.^43^ The first 16 substitutions enhance the enzyme thermostability and the E175A substitution relaxes the enzyme specificity for the NADP^+^ compared with the NAD^+^ cofactor. The protein was overexpressed and purified according to the published procedures.^44^ 17X-PTDH was exhaustively dialyzed against 36 mM TEA at pH 7.5 before use. The enzyme concentration was calculated after dialysis from the absorbance at 280 nm using a subunit molecular weight of 36400 Da and an extinction coefficient of ε = 27,000 M^–1^ cm^–1^ that was calculated using the ProtParam tool available on the ExPASy server.^45,46^

### Enzyme Kinetic Parameters

All enzyme assays were carried out at 25 °C using a Cary 3500 Multicell UV–vis spectrophotometer equipped with temperature-controlled Peltier block multicell changer. The concentrations of NADH, NRH and NMNH formed as products of enzyme-catalyzed-reduction of the oxidized cofactor or cofactor fragment were determined from the change in absorbance at 340 nm using, respectively, extinction coefficients of 6220, 4900,^47^ and 5160 M^–1^ cm^–1^.^48^ The enzyme kinetic parameters were obtained from the linear or nonlinear least-squares fits of the kinetic data to the appropriate kinetic equation using the Prism 10 program from GraphPad Software.

#### 17X-PsPTDH-Catalyzed Oxidation of HPO_3_^2–^ by NAD^+^

17X-*Ps*PTDH-catalyzed hydride transfer from HPO_3_^2–^ to NAD^+^ was monitored in 1.0 mL solutions that contain 36 mM TEA buffer (pH 7.5) at *I* = 0.3 (NaCl), 0.1 mg/mL BSA and either 1 mM NAD^+^ at increasing concentrations (0.005–16 mM) of HPO_3_^2–^ or 20 mM HPO_3_^2–^ at increasing concentrations (0.0025–2.00 mM) of NAD^+^. The reactions were initiated by addition of 17X-*Ps*PTDH to give final enzyme concentrations of *ca* 0.3 μM. The initial reaction velocity *v* (M/s) was determined from the increase in absorbance at 340 nm observed for 10 min reactions that consumed ≤ 10% of the limiting substrate.

#### 17X-PsPTDH-Catalyzed Oxidation of HPO_3_^2–^ by NR

The 17X-*Ps*PTDH-catalyzed oxidation of HPO_3_^2–^ (1–12 mM) by NR (1–10 mM) was monitored in 1.0 mL solutions that contained 36 mM TEA buffer (pH 7.5) at *I* = 0.3 (NaCl) and 0.1 mg/mL BSA. The reactions were initiated by addition of 17X-*Ps*PTDH to give the following final enzyme concentrations; 4.6 μM for reactions of 1.0 mM NR and 2.0 μM for reactions of 5.0 mM and 10.0 mM NR. The initial reaction velocity *v* (M/s) was determined by monitoring the increase in absorbance at 340 nm for 12 h reactions that consumed < 1% of NR.

#### Enzyme-Catalyzed Oxidation of HPO_3_^2–^ by NMN

The 17X-*Ps*PTDH-catalyzed oxidation of HPO_3_^2–^ (1–16 mM) by NMN (1–12 mM) was monitored in 1.0 mL solutions that contained 36 mM TEA buffer (pH 7.5) at *I* = 0.3 (NaCl) and 0.1 mg/mL BSA. The reactions were initiated by the addition of 17X-*Ps*PTDH to give a final enzyme concentration of 0.036 μM. The initial reaction velocity *v* (M/s) was determined by monitoring the increase in absorbance at 340 nm for 12 h reactions that consumed < 1% of NMN.

*Cb*FDH was screened for catalysis of hydride transfer from formate to NMN at 25 °C in a 1.0 mL solution that contained 36 mM TEA buffer (pH 7.5) at *I* = 0.3 (NaCl), 0.1 mg/mL BSA, 20 mM NMN, 150 mM formate and 30 μM *Cb*FDH. There was no change in absorbance at 340 nM (≤0.01 absorbance unit) observed over a 12 h reaction time. *hl*GPDH was screened for catalysis of hydride transfer from G3P to NMN at 25 °C in a 1.0 mL solution that contained 20 mM TEA buffer (pH 7.5) at *I* = 0.12 (NaCl), 0.1 mg/mL BSA, 20 mM NMN, 3 mM G3P and 110 μM *hl*GPDH. There was no change in absorbance at 340 nM (≤0.01 absorbance unit) observed over a 12 h reaction time.

#### hlGPDH-Catalyzed Reduction of NAD^+^ by G3P

The assay mixtures contained 20 mM TEA at pH 7.5 (*I* = 0.12, NaCl), 0.1 mg/mL BSA, L-G3P (10–50 μM), NAD^+^ (10–20 μM) and 2 nM *hl*GPDH. The initial reaction velocity *v* (M/s) was determined by monitoring the increase in absorbance at 340 nm for 10 min reactions that consumed ≤ 10% of the limiting substrate.

#### Fragment Activated 17X-PsPTDH-Catalyzed Hydride Transfer from HPO_3_^2–^ to NR

The reactions were monitored in 1.0 mL solutions that contained 36 mM TEA buffer (pH 7.5) at *I* = 0.3 (NaCl), 0.1 mg/mL BSA, NR (1–10 mM), HPO_3_^2–^ (4–16 mM) and the following ranges of activator concentrations; ADP (0.5–8 mM), AMP (0.5–16 mM), adenosine (8 mM). The reactions were initiated by the addition of 17X-*Ps*PTDH to give enzyme final concentrations that range from 1 to 3 μM. The initial reaction velocity *v* (M/s) was determined by monitoring the increase in absorbance at 340 nm for 12 h reactions that consumed < 10% of the limiting substrate.

## Results

This work was enabled by our use of the thermostable 17X-*Ps*PTDH construct of *Ps*PTDH that shows no detectable loss in catalytic activity during reaction times of up to 12 h at 25 °C. Figure S1 in the Supporting Information (SI) shows the mass spectrum for our preparation of 17X-*Ps*PTDH determined on a 6500 Q-TOF LC/MS. The spectrum was deconvoluted using BioConfirm software to give a major peak with a protein subunit molecular weight of 36764.1 Da. The mass is in a good agreement with the molecular weight for 17X-*Ps*PTDH (36764.4 Da) calculated using the ProtParam tool for a protein where the Gly-Ser-His tripeptide at the thrombin cleavage site remains attached to the N-terminal methionine.^45,46^

17X-*Ps*PTDH-catalyzed hydride transfer from HPO_3_^2–^ to NAD^+^ was monitored at pH 7.5 (36 mM TEA buffer) and a constant *I* = 0.3 maintained with NaCl. Figure S2 shows the dependence of *v*/[E] on [HPO_3_^2–^] for 17X-*Ps*PTDH-catalyzed reactions at a saturating concentration of 1.0 mM NAD^+^. The fit of the data from Figure S2 to the Michaelis–Menten equation gave the kinetic parameters *k*_cat_ = 2.3 ± 0.02 s^–1^ and *K*_HPi_ = 4.1 ± 0.1 mM. Figure S3 shows the dependence of *v*/[E] on [NAD^+^] for reactions at a constant nearly saturating concentration of 20 mM HPO_3_^2–^. The fit of the data from Figure S3 to the Michaelis–Menten equation gave the kinetic parameters *k*_cat_ = 2.1 ± 0.02 s^–1^ and *K*_NAD_ = 0.082 ± 0.003 mM. Combining values for *k*_cat_/*K*_HPi_ = 560 M^–1^ s^–1^ and *K*_NAD_ = 8.2 × 10^–5^ M gives the apparent third-order rate constant *k*_cat_/*K*_NAD_ *K*_HPi_ = (6.8 ± 0.3) × 10^6^ M^–2^ s^–1^ reported in Table 1. By comparison, values of *k*_cat_ = 3.3 s^–1^, *K*_HPi_ = 0.028 mM and *K*_NAD_ = 0.022 mM were reported in an earlier study on 17X-*Ps*PTDH-catalyzed hydride transfer at pH 7.25 (100 mM MOPS) and at a lower and variable ionic strength.^43^ We attribute the large difference in the values of *K*_HPi_ = 4.1 mM determined here for reactions at constant *I* = 0.3 and *K*_HPi_ = 0.028 mM determined at low ionic strength to the effect of NaCl on the activity (*a* = γ­[ HPO_3_^2–^]) of phosphite dianion. A similar increase with increasing ionic strength has been reported for the Michaelis constants *K*_m_ for orotidine 5′-monophosphate determined for decarboxylation reactions catalyzed by wild-type and variants of OMPDC.^49,50^

**Table 1:** Rate Constants for Unactivated and Activated 17X-*Ps*PTDH-Catalyzed Hydride Transfer from HPO_3_^2–^ to the Whole Cofactor NAD^+^ and the Truncated Cofactors NR and NMN^a^

hydride donor	hydride acceptor	activator	rate constant
HPO_3_^2–^	NAD^+^	none	*k*_cat_/*K*_NAD_ *K*_HPi_ 6.8 × 10^6^ M^–2^ s^–1^ ^b^
			*k*_cat_/*K*_ia_ *K*_HPi_ (1.35 ± 0.03) × 10^6^ M^–2^ s^–1^ ^c^
	NMN		*k*_cat_/*K*_NMN_ *K*_HPi_ 310 ± 6 M^–2^ s^–1^ ^d^
	NR		*k*_cat_/*K*_NR_ *K*_HPi_ 0.78 ± 0.01 M^–2^ s^–1^ ^e^
	NR	ADP	*k*_cat_^′^/*K*_NR_ *K*_Act_ *K*_HPi_ 4400 ± 100 M^–3^ s^–1^ ^f^
	NR	AMP	*k*_cat_^′^/*K*_NR_ *K*_Act_ *K*_HPi_ 70 ± 1.3 M^–3^ s^–1^ ^g^

a For reactions at 25 °C, pH 7.5 (36 mM TEA buffer) and *I* = 0.3 (NaCl). Quoted uncertainty is the standard deviation of the slope of the relevant linear plot.

b Third-order rate constant determined as described in the text.

c Slope of the linear plot from Figure 1.

d The slope of the linear plot from Figure 3.

e Slope of the linear plot from Figure 2.

f Slope of the linear plot from Figure 5B.

g Calculated from the slope of the linear plot from Figure 6B with the assumption that *k*′_cat_/*K*_NR_ *K*_Act_ *K*_HPi_ = (*k*′_cat_/*K*_NR_ *K*_Act_)_obs_/[HPO_3_^2–^].

Figure 1 shows the increase in *v*/[E] with increasing [HPO_3_^2–^]­[NAD^+^] for 17X-*Ps*PTDH-catalyzed hydride transfer at pH 7.5 (36 mM TEA buffer) and a constant *I* = 0.3 maintained with NaCl, where [NAD^+^] ≪ *K*_NAD_ = 0.082 mM and [HPO_3_^2–^] ≪ *K*_HPi_ = 4.1 mM. The slope of this plot is the third-order rate constant *k*_cat_/*K*_ia_ *K*_HPi_ = (1.35 ± 0.03) × 10^6^ M^–2^ s^–1^ (Table 1). Figure 2 shows the increase in *v*/[E] with increasing [HPO_3_^2–^]­[NR] for 17X-*Ps*PTDH-catalyzed hydride transfer from HPO_3_^2–^ to the truncated cofactor NR at pH 7.5 (36 mM TEA buffer) and a constant *I* = 0.3 maintained with NaCl. The slope of this linear correlation is the third-order rate constant *k*_cat_/*K*_NR_ *K*_HPi_ = 0.78 ± 0.01 M^–2^ s^–1^ (Scheme 3 and Table 1).

**Figure 1:**
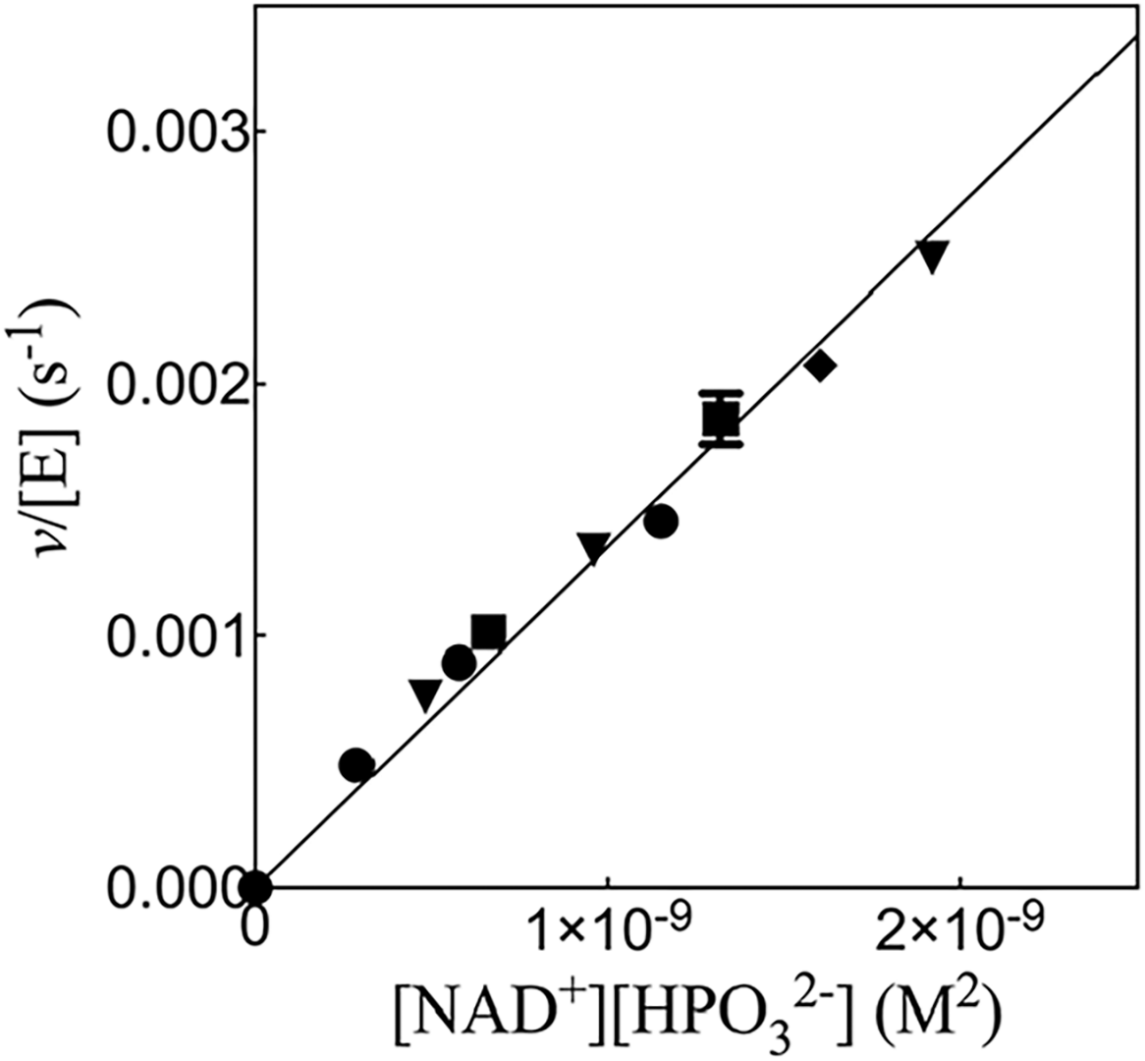
Effect of the increasing [NAD^+^]­[HPO_3_^2–^] on *v*/[E] for 17X-*Ps*PTDH-catalyzed hydride transfer at [HPO_3_^2–^] ≪ *K*_HPi_ = 4.1 mM (Figure S2) and [NAD^+^] ≪ *K*_NAD_ = 0.082 mM (Figure S3). Key: [NAD^+^] = 10 μM, circles: [NAD^+^] = 13 μM, diamond; [NAD^+^] = 16 μM, inverted triangles; [NAD^+^] = 22 μM, squares. The error bars show the range of values for duplicate determinations of *v*/[E].

**Figure 2:**
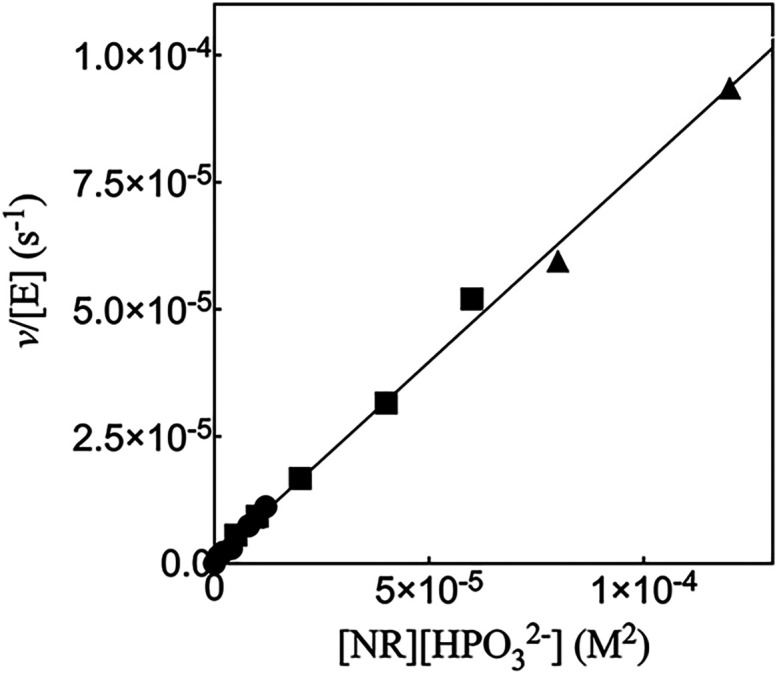
Effect of the increasing [NR]­[HPO_3_^2–^] on *v*/[E] for 17X-*Ps*PTDH-catalyzed hydride transfer. Key: [NR] = 1 mM, solid circles; [NR] = 5 mM, solid squares; [NR] = 10 mM, solid triangles.

**Scheme 3:**
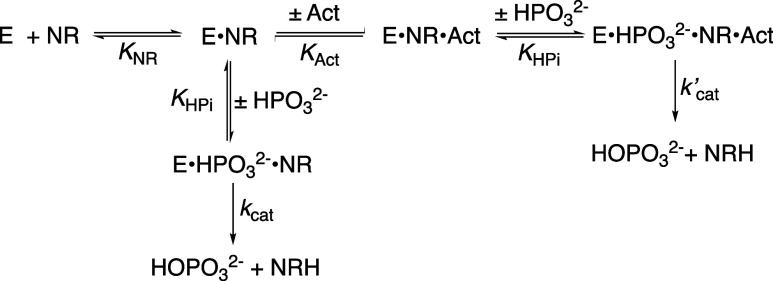
Kinetic Mechanism for Unactivated and Activated 17X-*Ps*PTDH-Catalyzed Hydride Transfer from HPO_3_^2–^ to NR to Form HOPO_3_^2–^ and NRH

Figure 3 shows the increase in *v*/[E] with increasing [HPO_3_^2–^]­[NMN] for 17X-*Ps*PTDH-catalyzed hydride transfer from HPO_3_^2–^ to the truncated cofactor NMN at pH 7.5 (36 mM TEA buffer) and a constant *I* = 0.3 maintained with NaCl. The slope of this linear correlation is equal to the third-order rate constant *k*_cat_/*K*_NMN_ *K*_HPi_ = 310 ± 6 M^–2^ s^–1^ (Table 1). *Cb*FDH and *hl*GPDH were screened for activity as catalysts of hydride transfer from their respective substrates to NMN at enzyme and cofactor analog concentrations similar to those used to determine the kinetic parameters for catalysis of hydride transfer to NR.^30,31^ No NMNH was detected from a 12 h reaction of 150 mM formate with 20 mM NMN in the presence of 30 μM *Cb*FDH or from a 12 h reaction of 3 mM G3P with 20 mM NMN in the presence of 110 μM *hl*GPDH. We conclude that *Cb*FDH and *hl*GPDH are less effective catalysts of hydride transfer to NMN compared to NR, for which values of *k*_cat_/*K*_NR_ *K*_F_ = 0.0026 M^–2^ s^–1^ and *k*_cat_/*K*_NR_ *K*_G3P_ = 0.058 M^–2^ s^–1^ have been reported.^30,31^

**Figure 3:**
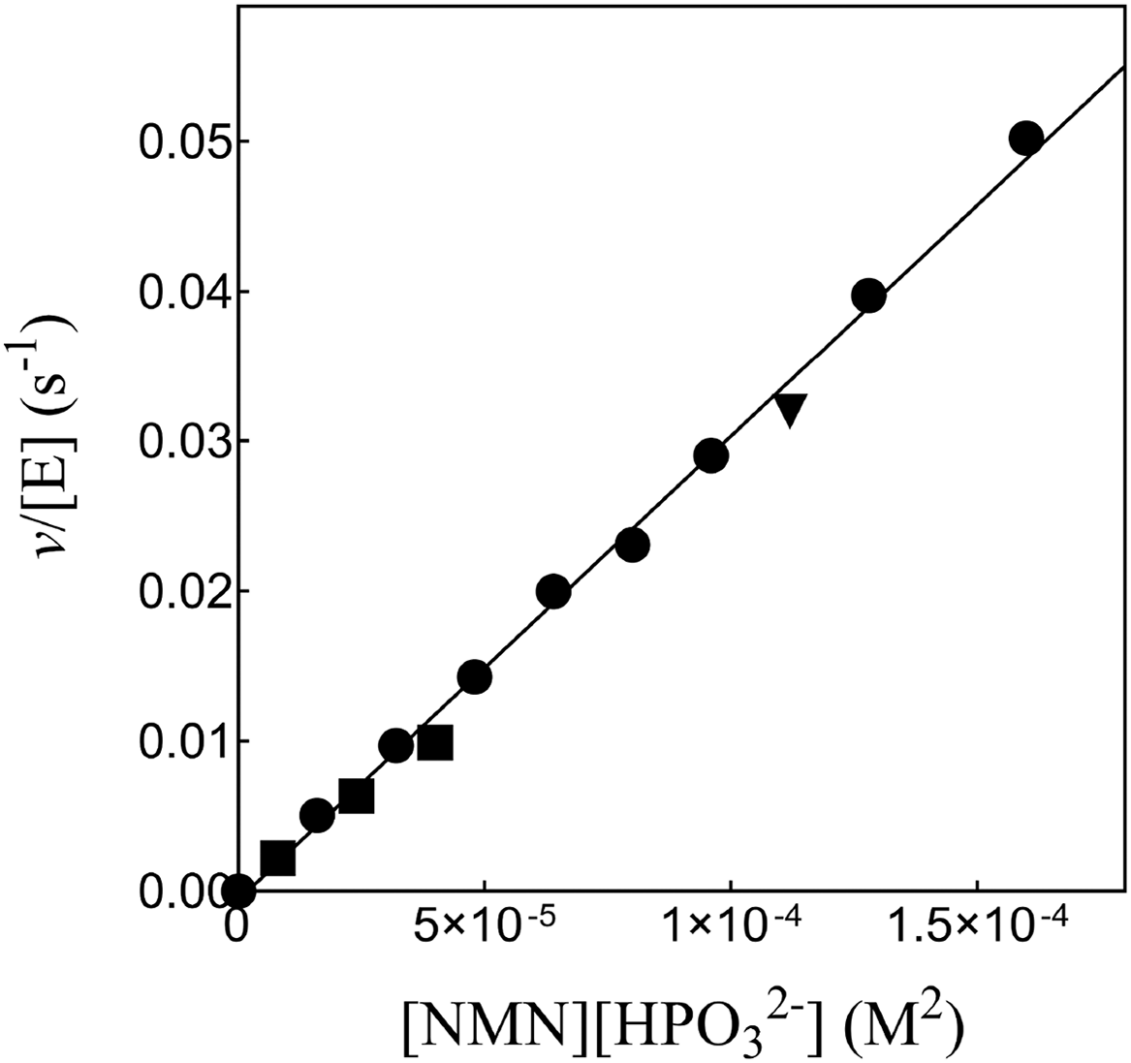
Effect of increasing [NMN]­[ HPO_3_^2–^] on *v*/[E] for 17X-*Ps*PTDH-catalyzed hydride transfer. Key: [HPO_3_^2–^] = 16 mM, solid circles; [HPO_3_^2–^] = 4 mM, solid squares; [HPO_3_^2–^] = 8 mM, solid triangle.

Figure 4 shows the increase in *v*/[E] with increasing concentrations of ADP activator for 17X-*Ps*PTDH-catalyzed hydride transfer reactions of 4 mM phosphite dianion at different fixed concentrations of the truncated cofactor NR, where *v*_0_/[E] is the value observed in the absence of ADP activator. The data from Figure 4 for reactions at different [NR] were fit to eq 1 to give values for the apparent second-order rate constants (*k*′_cat_/*K*_Act_)_obs_ (eq 2) for reactions at low [ADP]. Figures S4 and S5 show, respectively, the increase in *v*/[E] with increasing concentrations of ADP activator for 17X-*Ps*PTDH-catalyzed hydride transfer reactions of 8 mM and 16 mM phosphite dianion at different fixed concentrations of NR. The data from Figures S4 and S5 for reactions at different [NR] were fit to eq 1 to give values for the observed second-order rate constants (*k*_cat_/*K*_Act_)_obs_ (eq 2) for reactions at different fixed concentrations of NR.

1 $$\frac{v}{\left[E\right]}=\frac{v_{o}}{\left[E\right]}+\frac{k_{\mathrm{cat}}^{\prime }\left[\mathrm{Act}\right]\left[\mathrm{NR}\right]\left[{HPO_{3}}^{2-}\right]}{K_{\mathrm{NR}}K_{\mathrm{Act}}K_{\mathrm{HPi}}+K_{\mathrm{HPi}}\left[\mathrm{Act}\right]\left[\mathrm{NR}\right]}$$

2 $${\left(\frac{k^{\prime }}{K_{\mathrm{act}}}\right)}_{\mathrm{obs}}=\frac{k_{\mathrm{cat}}^{\prime }\left[\mathrm{NR}\right]\left[{HPO_{3}}^{2-}\right]}{K_{\mathrm{NR}}K_{\mathrm{Act}}K_{\mathrm{HPi}}}$$

3 $${\left(\frac{k^{\prime }}{K_{\mathrm{NR}}K_{\mathrm{act}}}\right)}_{\mathrm{obs}}=\frac{k_{\mathrm{cat}}^{\prime }\left[{HPO_{3}}^{2-}\right]}{K_{\mathrm{NR}}K_{\mathrm{Act}}K_{\mathrm{HPi}}}$$

Figure 5A shows the effect of increasing [NR] on the values of (*k*′_cat_/*K*_Act_)_obs_ (M^–1^ s^–1^) determined for Figures 4, S4 and S5. The slopes of these correlations (eq 2) are equal to the apparent third-order rate constants (*k*′_cat_/*K*_NR_ *K*_Act_)_obs_ (M^–2^ s^–1^, eq 3) for ADP-activated 17X-*Ps*PTDH-catalyzed hydride transfer determined at different fixed [HPO_3_^2–^]. Figure 5B shows the effect of increasing concentrations of HPO_3_^2–^ on the third-order rate constants (*k*′_cat_/*K*_NR_ *K*_Act_)_obs_. The slope of this linear correlation is the fourth-order rate constant *k*′_cat_/*K*_NR_ *K*_Act_ *K*_HPi_ = 4400 ± 100 M^–3^ s^–1^ (eq 3) for ADP activated 17X-*Ps*PTDH-catalyzed hydride transfer from HPO_3_^2–^ to NR (Table 1).

**Figure 4:**
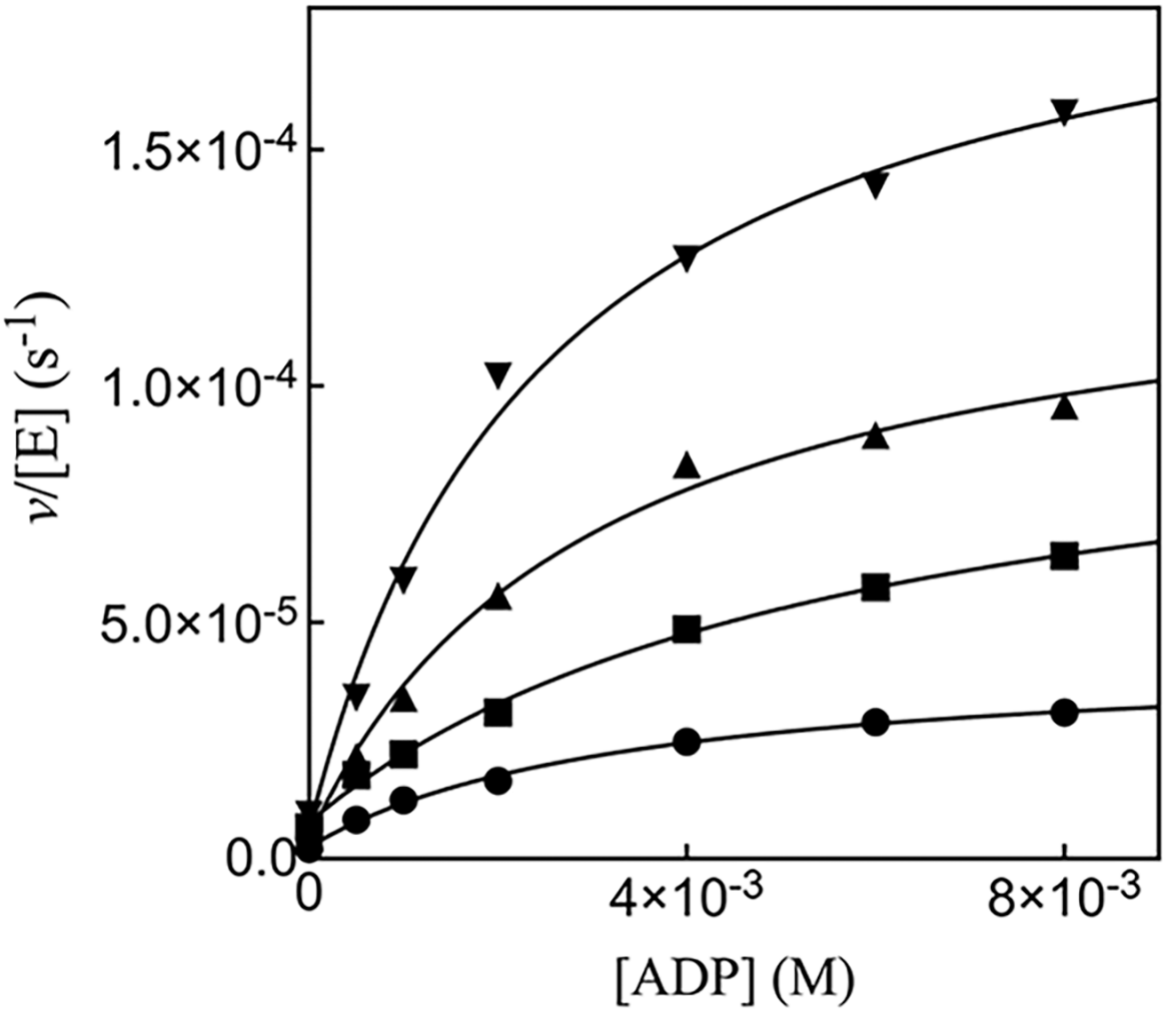
Effect of increasing [ADP] on *v*/[E] for 17X-*Ps*PTDH-catalyzed hydride transfer from 4 mM phosphite dianion to different fixed concentrations of NR. Key: 1 mM NR, circles, 2 mM NR, squares, 3 mM NR, triangles, 5 mM NR, inverted triangles.

**Figure 5:**
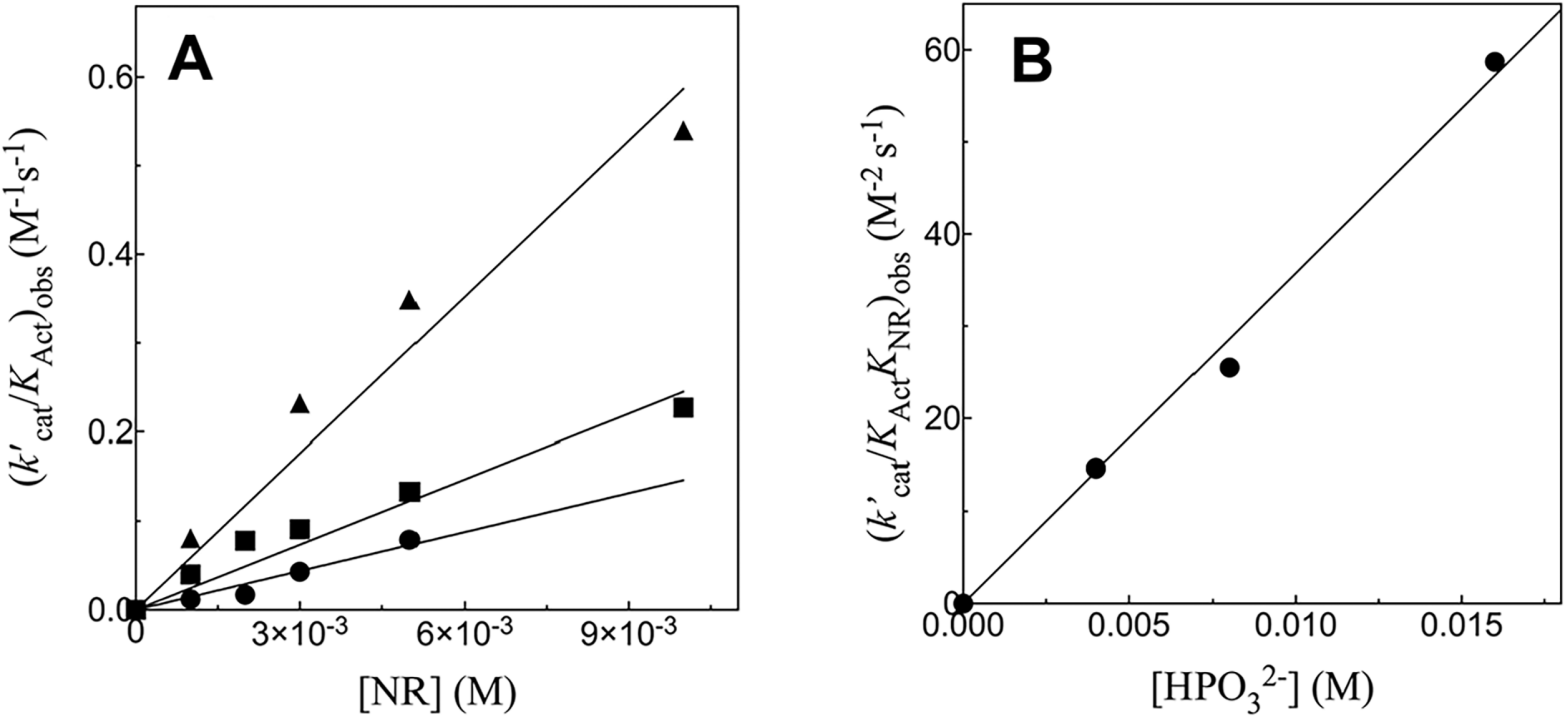
(A) The effect of increasing [NR] on the apparent second-order rate constants (*k*_cat_/*K*_act_)_obs_ for ADP-activated 17X-*Ps*PTDH-catalyzed hydride transfer reactions determined for data from Figures 4, S4 and S5: Key: 4 mM HPO_3_^2–^, circles (data from Figure 4); 8 mM HPO_3_^2–^ squares (data from Figure S4); 16 mM HPO_3_^2–^, triangles (data from Figure S5). (B) The effect of increasing [HPO_3_^2–^] on the apparent third-order rate constants (*k*′_cat_/*K*_Act_ *K*_NR_)_obs_ determined as the slopes of the correlations from Figure 5A.

Figure 6A shows the increase in *v*/[E] with increasing [AMP] for 17X-*Ps*PTDH-catalyzed hydride transfer reactions of 16 mM phosphite dianion at different fixed concentrations of NR, where *v*_0_/[E] is the value observed in the absence of ADP activator. The slopes of the individual linear correlations are equal to (*k*′_cat_/*K*_Act_)_obs_ for activation at the given [NR]. Figure 6B shows the increase in (*k*′_cat_/*K*_Act_)_obs_ with increasing [NR] for AMP activation of 17X-*Ps*PTDH-catalyzed hydride transfer reactions using data from Figure 6A. The slope of this linear correlation is equal to the third-order rate constant (*k*′_cat_/*K*_NR_ *K*_Act_)_obs_ = 1.06 ± 0.02 M^–2^ s^–1^ for reactions at 16 mM phosphite dianion. If the observed third-order rate constants for activation by AMP are first-order in the concentration of phosphite dianion (see Figure 5B for activation by ADP), then *k*′_cat_/*K*_NR_ *K*_Act_ *K*_HPi_ = (*k*′_cat_/ *K*_NR_ *K*_Act_)_obs_/[HPO_3_^2–^] = 1.06 M^–2^ s^–1^/0.016 M = 70 M^–3^ s^–1^ (Table 1). There is no detectable activation of 17X-*Ps*PTDH-catalyzed (3 μM) hydride transfer from phosphite dianion (16 mM) to NR (10 mM) observed for a reaction in the presence of 8 mM adenosine.

**Figure 6:**
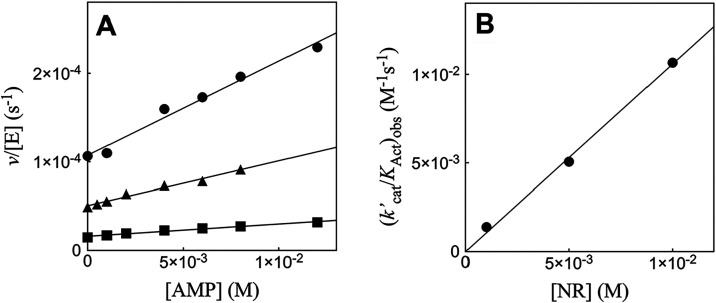
(A) The effect of increasing [AMP] on *v*/[E] for 17X-*Ps*PTDH-catalyzed hydride transfer from 16 mM phosphite dianion to different fixed concentrations of NR. Key: 1 mM NR, squares; 5 mM NR, triangles; 10 mM NR, circles. (B) The effect of increasing [NR] on the apparent second-order rate constant (*k*′_cat_/*K*_Act_)_obs_ for activation by AMP determined as the slopes of the correlations from Figure 6A.

Figure 7 shows the increase in *v*/[E] with increasing [G3P]­[NAD^+^] for *hl*GPDH-catalyzed hydride transfer at 25 °C, pH 7.5 (20 mM TEA) and *I* = 0.12 (NaCl), where [NAD^+^] ≪ *K*_NAD_ = 0.21 mM and [G3P] ≪ *K*_HPi_ = 0.19 mM. The slope of this plot is the third-order rate constant *k*_cat_/*K*_ia_ *K*_G3P_ = (9.7 ± 0.1) × 10^7^ M^–2^ s^–1^. By comparison, a value of *k*_cat_/*K*_NAD_ *K*_G3P_ = 4.3 × 10^8^ M^–2^ s^–1^ was determined for *hl*GPDH, where *K*_NAD_ and *K*_G3P_ are Michaelis constants for NAD^+^ and G3P (see Discussion).^30,51^

**Figure 7:**
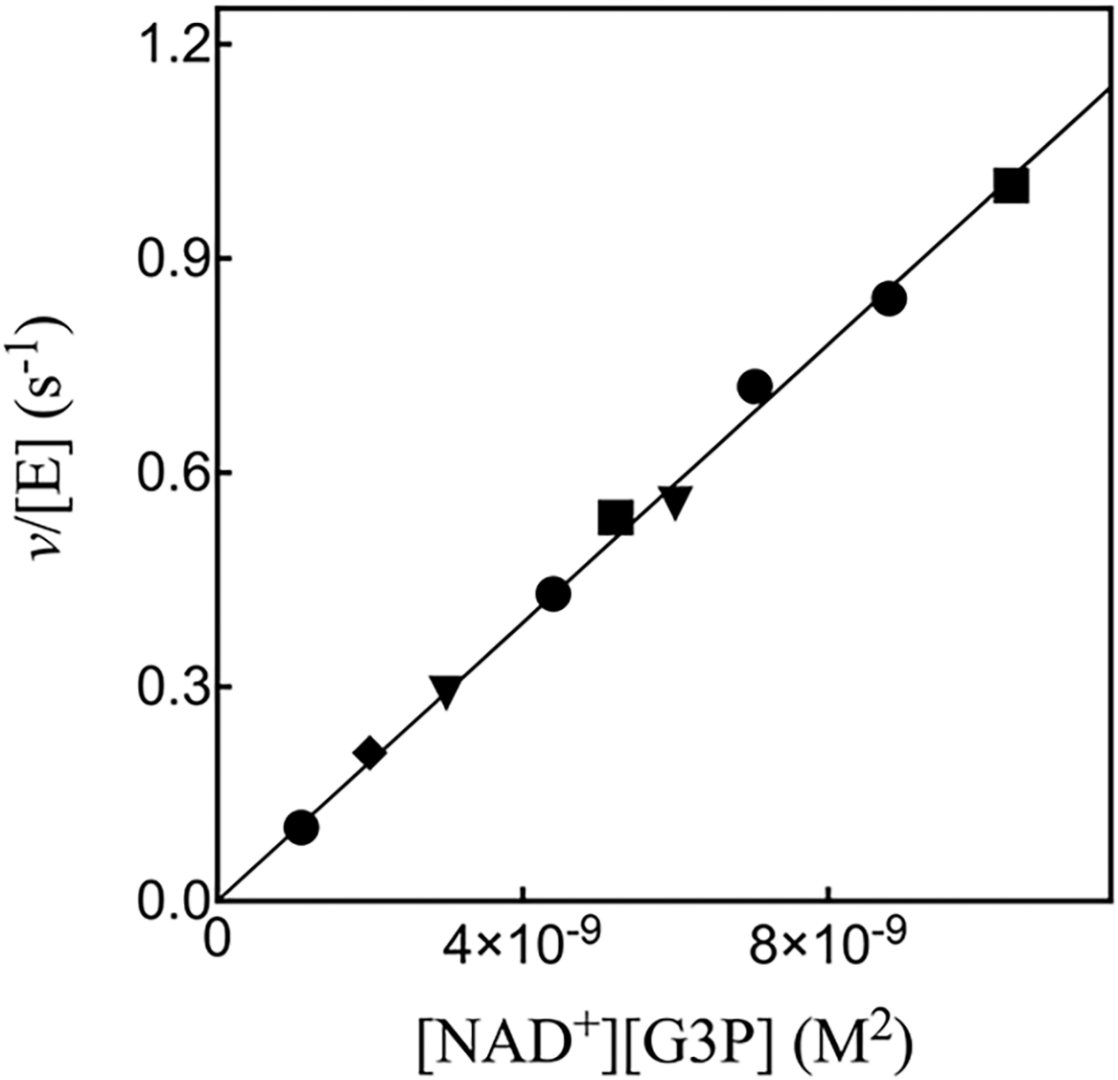
Effect of the increasing [NAD^+^]­[G3P] on *v*/[E] for *hl*GPDH-catalyzed hydride transfer at [G3P] ≪ *K*_G3P_ = 0.19 mM and [NAD^+^] ≪ *K*_NAD_ = 0.21 mM.^30,51^ Key: [NAD^+^] = 40 μM, diamond; [NAD^+^] = 44 μM, circles; [NAD^+^] = 50 μM, triangles; [NAD^+^] = 90 μM, squares.

## Discussion

The steady state kinetic and presteady state kinetic parameters, kinetic isotope effects and pH dependence for 17X-*Ps*PTDH are similar to those for the wild-type enzyme from *P. stutzeri*.^52^ No significant differences were noted in the positioning of active site amino acid side chains observed for the X-ray crystal structures of wild-type *Ps*PTDH and 17X-*Ps*PTDH.^44^

*Ps*PTDH follows an ordered bi-bi kinetic mechanism (Scheme 4) with the NAD^+^ cofactor binding before HPO_3_^2–^.^39^ The full steady-state rate equation for the ordered bi-bi reaction of NAD^+^ and HPO_3_^2–^ (eq 4)^53^ collapses to *v*/[E] = *k*_cat_[HPO_3_^2–^]­[NAD^+^]/*K*_ia_ *K*_HPi_ for reactions at [NAD^+^] ≪ *K*_NAD_ < *K*_ia_ and [HPO_3_^2–^] ≪ *K*_HPi_. Under these conditions, the slope of the correlation of *v*/[E] against [HPO_3_^2–^]­[NAD^+^] (Figure 1) is equal to *k*_cat_/*K*_ia_ *K*_HPi_ = 1.35 × 10^6^ M^–2^ s^–1^ (Table 1), where *K*_ia_ = *k*_2_/*k*_1_ is the thermodynamic disassociation constant for NAD^+^ and *K*_HPi_ is the Michaelis constant for phosphite dianion.^53^

4 $$\frac{v}{\left[E\right]}=\frac{k_{\mathrm{cat}}\left[{\mathrm{NAD}}^{+}\right]\left[{HPO_{3}}^{2-}\right]}{K_{\mathrm{ia}}K_{\mathrm{HPi}}+K_{\mathrm{HPi}}\left[{\mathrm{NAD}}^{+}\right]+K_{\mathrm{NAD}}\left[{HPO_{3}}^{2-}\right]+\left[{\mathrm{NAD}}^{+}\right]\left[{HPO_{3}}^{2-}\right]}$$

A different third-order rate constant of *k*_cat_/*K*_NAD_ *K*_HPi_ = 6.8 × 10^6^ M^–2^ s^–1^ for 17X-*Ps*PTDH-catalyzed hydride transfer is obtained using values of *k*_cat_/*K*_HPi_ = 560 M^–1^ s^–1^ (Figure S2) determined for 17X-*Ps*PTDH-catalyzed hydride transfer at saturating NAD^+^ ([NAD^+^] ≫ *K*_NAD_) and of *K*_NAD_ = 8.2 × 10^–5^ M (Figure S3) determined for 17X-*Ps*PTDH-catalyzed hydride transfer at saturating HPO_3_^2–^ ([HPO_3_^2–^] ≫ *K*_HPi_). The ratio of the two-third-order rate constants from Table 1 is equal to *K*_ia_/*K*_NAD_ = 5.0 and reflects the higher apparent affinity of NAD^+^ under steady-state compared with rapid equilibrium reaction conditions. The thermodynamic disassociation constant *K*_ia_ is larger than the Michaelis constant *K*_NAD_ because *K*_ia_ is for E and E·NAD^+^ at rapid chemical equilibrium, while *K*_NAD_ is determined for reactions at steady state where the fast, and effectively irreversible, conversion of E·NAD^+^ to E·NAD^+^· HPO_3_^2–^ (*k*_3_[HPO_3_^2–^] > *k*_2_) causes [E·NAD^+^]_ss_ to drop below [E·NAD^+^]_eq_. Under steady-state reaction conditions the concentration of [E·NAD^+^] at saturating NAD^+^ is significantly smaller than [E]_T_; and *K*_NAD_ < *K*_ia_ where *K*_NAD_ is the concentration of NAD^+^ at which *v*_obs_ is 50% of *V*_max_ for reactions at saturating [HPO_3_^2–^]. By comparison the values of *k*_cat_/*K*_ia_ *K*_G3P_ = 1.0 × 10^8^ M^–2^ s^–1^ (Figure 7) and *k*_cat_/*K*_NAD_ *K*_G3P_ = 4.2 × 10^8^ M^–2^ s^–1^ determined for *hl*GPDH-catalyzed hydride transfer^30^ gives *K*_ia_/*K*_NAD_ = 4.2 for this enzyme-catalyzed hydride transfer reaction, which also occurs by an ordered bi-bi kinetic reaction mechanism.^54^

**Scheme 4:**
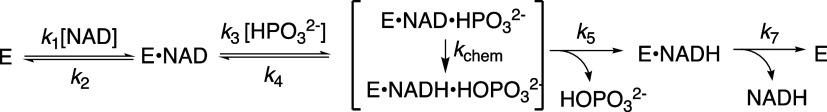
Ordered Reaction Mechanism for 17X-*Ps*PTDH-Catalyzed Hydride Transfer to NAD^+^

### 17X-*Ps*PTDH-Catalyzed Reactions of Truncated Cofactors

The third-order rate constants reported here and in earlier work^30,31^ for 17X-*Ps*PTDH-, *hl*GPDH- and *Cb*FDH-catalyzed hydride transfer reactions from reduced substrates SH to oxidized whole cofactor NAD^+^ or to truncated cofactors NMN and NR (Scheme 5) are summarized in Table 2. This Table shows interesting differences in the substrate specificity for 17X-*Ps*PTDH, compared with *Cb*FDH and *hl*GPDH.

**Scheme 5:**
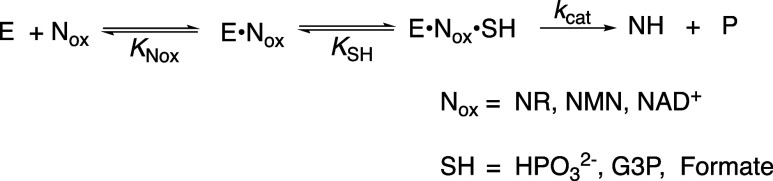
Kinetic Mechanism for Enzyme-Catalyzed Hydride Transfer from Reduced Substrates to the Oxidized Cofactor or Cofactor Fragment

**Table 2:** Rate Constants for Enzyme-Catalyzed Hydride Transfer Reactions of NAD^+^ and the Truncated Cofactors NR and NMN

		enzyme		
cofactor or cofactor fragment	kinetic parameter (M^–2^ s^–1^)	17X-*Ps*PTDH^a^	*hl*GPDH^b^	*Cb*FDH^c^
NR	*k*_cat_/*K*_NR_ *K*_SH_	0.78	0.058	0.0026
NMN	*k*_cat_/*K*_NMN_ *K*_SH_	310	<0.058^d^	<0.0026^d^
	Δ*G*_Pi_ ^e^	–3.5 kcal/mol	>0 kcal/mol	>0 kcal/mol
NAD^+^	*k*_cat_/*K*_NAD_ *K*_SH_	6.8 × 10^6^	4.2 × 10^8^	1.1 × 10^6^
	Δ*G*_ADP_^ss^ (kcal/mol)^f^	–9.5	–13.5	–11.7
	*k*_cat_/*K*_ia_ *K*_SH_	1.3 × 10^6^	9.7 × 10^7^	not determined
	Δ*G*_ADP_^eq^ (kcal/mol)^g^	–8.5	–12.5	not determined

a Data from Table 1.

b Data from ref 30.^30^

c Data from ref 31.^31^

d This work.

e Stabilization of the transition state for enzyme-catalyzed hydride transfer by interactions with the phosphate for NMN.

f Intrinsic binding energy of the ADP substrate fragment calculated from eq 5.

g Intrinsic binding energy of the ADP substrate fragment calculated from eq 6.

First, 17X-*Ps*PTDH shows a large specificity for hydride transfer to NMN (*k*_cat_/K_NMN_ *K*_SH_ = 310 M^–2^ s^–1^) compared to NR (*k*_cat_/*K*_NR_ *K*_SH_ = 0.78 M^–1^ s^–1^) that corresponds to a 3.5 kcal/mol stabilizing interaction between the enzyme and the NMN phosphate at a cofactor binding site that has evolved to stabilize the β-adenosyl phosphate of the NAD^+^ cofactor. By comparison, the observation that *k*_cat_/*K*_NMN_ *K*_SH_ < *k*_cat_/*K*_NR_ *K*_SH_ for *hl*GPDH- and *Cb*FDH-catalyzed hydride transfer (Table 2) is consistent with a different architecture of the cofactor binding site for these enzymes that gives rise to minimal stabilizing binding interactions with the NMN phosphate and, by analogy, with the β-adenosyl phosphate of NAD^+^.

Second, 17X-*Ps*PTDH shows 300-fold and 14-fold larger rate constants *k*_cat_/*K*_NR_ *K*_HPi_ for enzyme-catalyzed hydride transfer to NR, respectively, compared with hydride transfer reactions catalyzed by *hl*GPDH and *Cb*FDH. The larger activity of 17X-*Ps*PTDH for catalysis of hydride transfer to NR may be partly or entirely due to enzyme-activation by the substrate phosphite dianion at an activator site that also accepts the NMN phosphate or the β-adenosyl phosphate of the NAD^+^ cofactor.^5^ The action of phosphite dianion as both a substrate and activator predicts a quadratic dependence of *v*/[E] on [HPO_3_^2–^], and upward curvature for the plot of *v*/[E] against [HPO_3_^2–^]­[NR] (Figure 2). There is no sign of upward curvature for Figure 2, but this may be too small to detect by our methods.

### Intrinsic Binding Energy for the ADP Cofactor Fragment

The contribution of the ADP fragment from the NAD^+^ cofactor to the stabilization of the hydride transfer transition state for reactions catalyzed by 17X-*Ps*PTDH (SH = HPO_3_^2–^), *hl*GPDH (SH = G3P) or *Cb*FDH (SH = HCO_2_^–^) under steady-state and rapid equilibrium conditions (Δ*G*_ADP_^ss^ or Δ*G*_ADP_^eq^, Chart 1) were calculated from the ratio of rate constants for the enzyme-catalyzed reactions of the whole substrate NAD^+^ and the truncated substrate NR using either the rate constant *k*_cat_/*K*_NAD_ *K*_SH_ determined for reactions at high [SH] (Δ*G*_ADP_^ss^, eq 5) or *k*_cat_/*K*_ia_ *K*_SH_ determined for reactions at low [NAD^+^] ≪ *K*_NAD_ and [SH] ≪ *K*_SH_ (Δ*G*_ADP_^eq^, eq 6). The *ca* 1 kcal/mol difference between the values of Δ*G*_ADP_^ss^ and Δ*G*_ADP_^eq^ represents the kinetic advantage for the ordered reaction mechanism for enzyme-catalyzed reactions of NAD^+^ compared with the rapid equilibrium reaction mechanism for hydride transfer to NR, where the concentrations of both substrates are far below saturation. We use the values of Δ*G*_ADP_^eq^ from eq 6 for 17X-*Ps*PTDH and *hl*GPDH, because these were determined for experiments where the concentrations of both NAD^+^ and NR are held below their respective disassociation constants.

5 $$\Delta G_{\mathrm{ADP}}^{\mathrm{ss}}=-\mathrm{RT}\;\ln\left[\frac{\left(k_{\mathrm{cat}}/K_{\mathrm{NAD}}K_{\mathrm{SH}}\right)}{\left(k_{\mathrm{cat}}/K_{\mathrm{NR}}K_{\mathrm{SH}}\right)}\right]$$

6 $$\Delta G_{\mathrm{ADP}}^{\mathrm{eq}}=-\mathrm{RT}\;\ln\left[\frac{\left(k_{\mathrm{cat}}/K_{\mathrm{ia}}K_{\mathrm{SH}}\right)}{\left(k_{\mathrm{cat}}/K_{\mathrm{NR}}K_{\mathrm{SH}}\right)}\right]$$

The values of Δ*G*_ADP_ from Chart 1 show that 17X-*Ps*PTDH, *hl*GPDH and *Cb*FDH each obtain a large catalytic advantage from utilization of the cofactor-fragment binding energy in transition state stabilization. The apparent intrinsic binding energy Δ*G*_ADP_^eq^ = −8.5 kcal/mol calculated for 17X-*Ps*PTDH-catalyzed reactions at low substrate concentrations where E and E·NAD are at chemical equilibrium is 4 kcal/mol smaller than for *hl*GPDH (Table 2). However, the value of Δ*G*_ADP_^eq^ for 17X-*Ps*PTDH will underestimate the total fragment binding energy if, as discussed above, the kinetic parameter *k*_cat_/*K*_NR_ *K*_HPi_ includes a contribution from phosphite dianion activation, at the cofactor binding site, of enzyme-catalyzed hydride transfer to NR.

**Chart 1:**
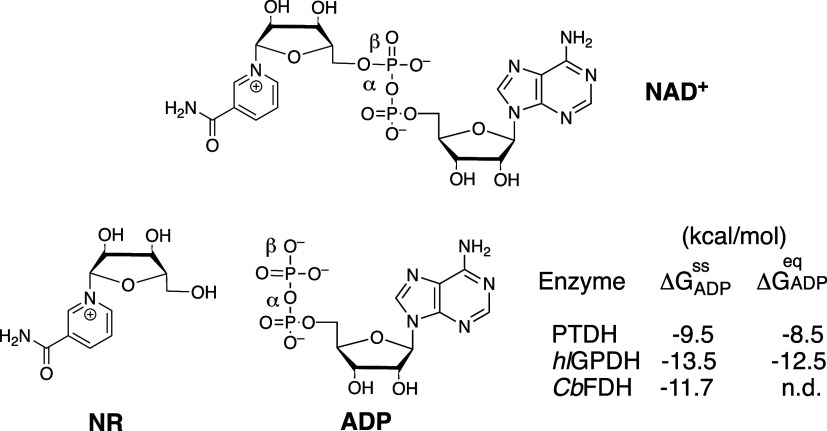
Intrinsic Binding Energy Δ*G*_ADP_^ss^ or Δ*G*_ADP_^eq^ for the ADP Cofactor Fragment Determined for Reactions Catalyzed by 17X-*Ps*PTDH, *hl*GPDH,^30^ and C*b*FDH^31^ from Kinetic Parameters Reported in Table 2 and Using Either eqs 5 or 6

### Activation of Enzyme-Catalyzed Hydride Transfer to NR by Cofactor Pieces

17X-*Ps*PTDH-, *hl*GPDH- and *Cb*FDH-catalyzed hydride transfer to the truncated NR cofactor are activated by addition of cofactor pieces.^30,31^ The fourth-order rate constants *k*_cat_^′^/*K*_NR_ *K*_Act_ *K*_SH_ (SH = HPO_3_^2–^, G3P, formate, Scheme 6) for activation of these enzymes by AMP and ADP are summarized in Table 3. The ratio of rate constants for fragment-activated and unactivated (*k*_cat_/*K*_NR_ *K*_SH_, Table 3) enzyme-catalyzed hydride transfer to NR define the values for 1/*K*_Act_^†^ and Δ*G*_act_^†^ (eqs 7 and 8 derived for Scheme 6) for activator binding to the transition state for the unactivated hydride transfer reaction. The activator binding energies for 17X-*Ps*PTDH, *Cb*FDH and *hl*GPDH are summarized in Chart 2. We note that the ADP activator binding energies from Table 3 may underestimate the true transition state stabilization by the ADP fragment of NAD^+^ because of destabilizing steric or electrostatic interactions between the −CH_2_OH group of NR and the β-phosphate of ADP.^31,55,56^

7 $$\frac{k_{\mathrm{cat}}^{\prime }/K_{\mathrm{NR}}K_{\mathrm{SH}}K_{\mathrm{Act}}}{k_{\mathrm{cat}}/K_{\mathrm{NR}}K_{\mathrm{SH}}}=\frac{k_{\mathrm{cat}}^{\prime }}{k_{\mathrm{cat}}K_{\mathrm{Act}}}=1/K_{\mathrm{Act}}^{†}$$

8 $$\Delta G_{\mathrm{act}}^{†}=-\mathrm{RT}\;\ln\left(1/K_{\mathrm{Act}}^{†}\right)$$

**Scheme 6:**
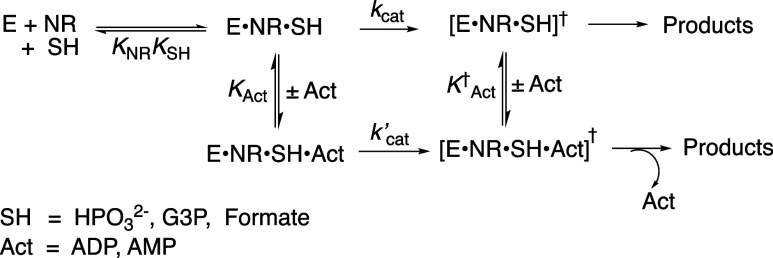
Unactivated (*k*_cat_) and Activated (*k*′_cat_) 17X-*Ps*PTDH-Catalyzed Hydride Transfer to NR

**Chart 2:**
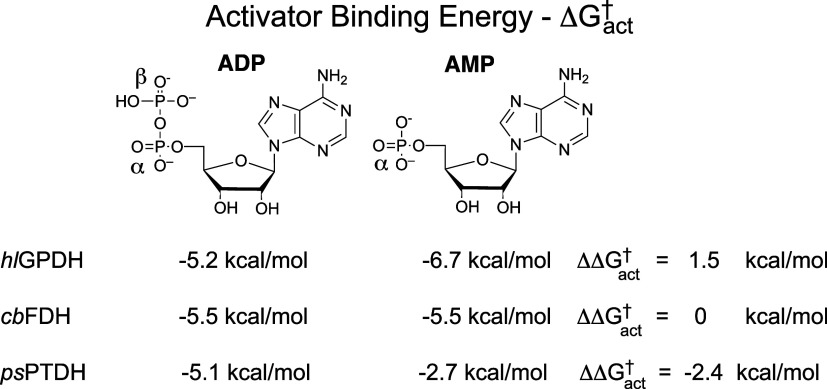
Transition State Stabilization for *hl*GPDH-, *Cb*FDH- and 17X-*Ps*PTDH-Catalyzed Hydride Transfer to NR by the ADP and AMP Fragments of the NAD^+^ Cofactor

**Table 3:** Rate Constants for Fragment Activated Enzyme-Catalyzed Hydride Transfer Reactions to NR and Fragment Intrinsic Binding Energies

	17X-*Ps*PTDH^a^	C*b*FDH^b^	*h*lGPDH^c^
activator	*k*_cat_^′^/*K*_NR_ *K*_Act_ *K*_SH_ (M^–3^ s^–1^)		
ADP	4400	30	400
Δ*G*_Act_^†^ (kcal/mol)^d^	–5.1	–5.5	–5.2
AMP	70	30	5100
Δ*G*_Act_^†^ (kcal/mol)^d^	–2.7	–5.5	–6.7

a Data from Table 1.

b Data from ref^30^

c Data from ref^31^

d Calculated using eqs 7 and 8.

Chart 2 shows that AMP stabilizes the transition state for *hl*GPDH-catalyzed hydride transfer to NR by 6.7 kcal/mol,^30^ for *Cb*FDH-catalyzed hydride transfer by 5.5 kcal/mol,^31^ and for 17X-*Ps*PTDH-catalyzed hydride transfer by 2.5 kcal/mol. The β-phosphate at ADP reduces by 1.5 kcal/mol this transition state stabilization for *hl*GPDH-catalyzed hydride to NR, does not affect transition state stabilization for *Cb*FDH-catalyzed hydride transfer and enhances stabilization by 2.5 kcal/mol for 17X-*Ps*PTDH-catalyzed hydride transfer. We conclude that fragment activation of *hl*GPDH- and *Cb*FDH-catalyzed hydride transfer is due largely to enzyme interactions with the AMP-cofactor fragment of NAD^+^, but that activation of *Ps*PTDH-catalyzed hydride transfer uses enzyme interactions with both the α- and β- ADP phosphates. There is no detectable activation of 17X-*Ps*PTDH-catalyzed hydride transfer to NR by 8 mM adenosine, so that fragment activation by ADP is due mainly to interactions with the pyrophosphate oxygen anions.

### Mechanism for Enzyme Activation

Scheme 7 provides a rationalization for the many observations of enzyme-activation by substrate-driven enzyme conformational changes,^4–6^ where the binding energy of nonreacting substrate fragments is utilized to drive changes in enzyme conformation from the dominant flexible inactive form **E**_**O**_ for unliganded enzyme, to the rigid, ordered, catalytically active conformation form **E**_**C**_ for the substrate Michaelis complex (*K*_C_ ≪1). We propose that dehydrogenase-activation by the AMP and ADP fragments of the NAD^+^ cofactor likewise arises from protein-activator interactions that stabilize the active closed enzyme **E**_**C**_ relative to **E**_**O**_.

**Scheme 7:**
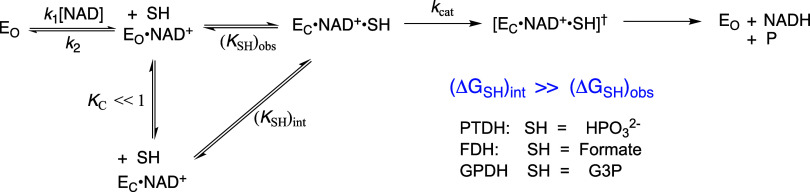
Induced-Fit Mechanism for Hydride Transfer Catalyzed by *Cb*FDH, *hl*GPDH and 17X-*Ps*PTDH

The values of Δ*G*_act_^†^ from Chart 2 show that activation of *Cb*FDH and *hl*GPDH is from interactions between the protein catalysts and the AMP cofactor fragment and that there are minimal stabilizing protein interactions with the β-phosphate of ADP. These conclusions were supported by an examination of the X-ray crystal structures for the unliganded and liganded forms of *Cb*FDH^20^ and *hl*GPDH,^12^ as discussed in earlier reports of fragment activation of these enzymes.^30,31^ By comparison, the activator binding energies for 17X-*Ps*PTDH (Chart 2) require that the closed enzyme **E**_**C**_ be stabilized relative to **E**_**O**_ by protein interactions with both the α- and β-phosphates of ADP. The absence of activation by adenosine shows that the open and closed forms of 17X-*Ps*PTDH show similar interactions with the adenosyl fragment of NAD^+^.

Figure 8 shows representations of the X-ray crystal structure for the open form of 17X-*Ps*PTDH (PDB entry 4EBF) and Figure 9 shows representations of the structure for the ternary **E**_**C**_·**NAD** ^**+**^·**SO**_**3**_^**2–**^ complex to the closed form of 16X-*Ps*PTDH [PDB entry 4E5K], where 16X-*Ps*PTDH is missing the E175A substitution at 17X-*Ps*PTDH that relaxes the NAD^+^ cofactor specificity for wild-type *Ps*PTDH.^44^ The positions of the NAD^+^ and SO_3_^2–^ ligands were modeled at Figure 8 by superimposing the structures from PDB entries 4EBF and 4E5K and then dissolving all of 4E5K except for the enzyme-bound ligands. The following observations from Figures 8 and 9 provide a structure-based rationale for the observations that the α- and β-phosphates of the ADP-cofactor fragment activate *Ps*PTDH for catalysis of hydride transfer and that there is no detectable activation by adenosine.

(1) There are only weak interactions between the protein and the α- and β-phosphates of ADP at the open enzyme (Figure 8). The cofactor binding induces a ∼ 6° rotation of the hinge that connects the substrate and cofactor domains and a narrowing of the active site cleft.^44^ This gives rise to movement of the flexible A74-D82 loop (red, Figures 8 and 9) toward the bound cofactor that is driven by formation of stabilizing ion-pairing interactions with the K76 side chain cation (violet, Figures 8 and 9) that bridge the α–and β–phosphates of NAD^+^.^44^ These interactions develop during the conformational change from **E**_**O**_ to **E**_**C**_ and activate the enzyme for catalysis of hydride transfer.
(2) Figure 8A shows that direct binding of NAD^+^ at the cleft for the cofactor leaves the cofactor adenosyl fragment exposed to solvent; this fragment remains exposed to solvent at the ternary complex (Figure 9A). We conclude that protein interactions with the fragment are effectively the same at the open enzyme **E**_**O**_ and the closed enzyme **E**_C_ (Scheme 7) and therefore do not contribute to ADP activation of 17X-*Ps*PTDH-catalyzed hydride transfer to NR.

**Figure 8:**
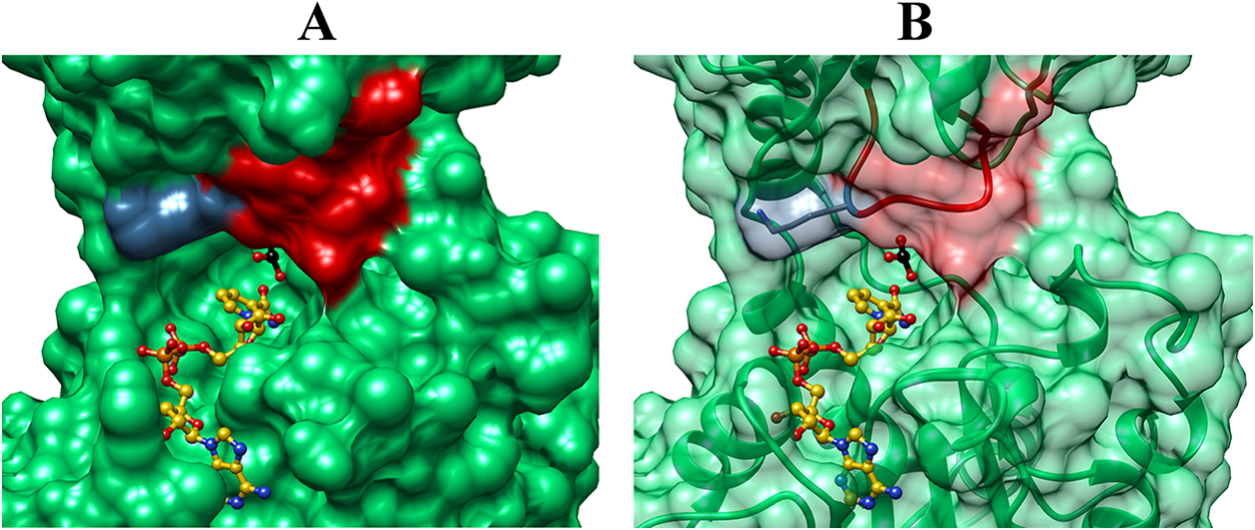
Representations of the X-ray crystal structure for unliganded 17X-*Ps*PTDH [PDB entry 4EBF] that show the NAD^+^ cofactor and sulfite dianion (sulfur colored black) modeled into the positions occupied at the ternary complex.^44^ (A) NAD^+^ sits on a cleft at the enzyme and is distant from the catalytic K76 side chain (violet) which is part of the A74-D82 loop (red). (B) The K76 side chain (violet) is shown at the A74-D82 loop (red ribbon).

**Figure 9:**
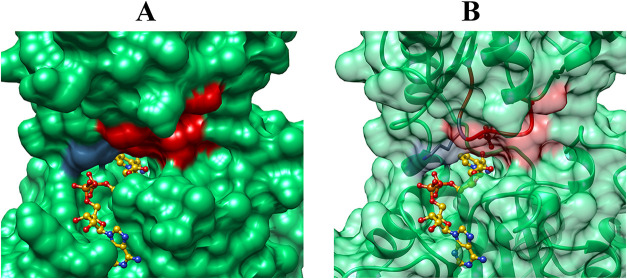
Representations of the X-ray crystal structure for the ternary complex of NAD^+^ and sulfite dianion to 16X-*Ps*PTDH [PDB entry 4E5K],^44^ which has 16 of the 17 substitutions for 17X-*Ps*PTDH but is missing the E175A substitution that relaxes the NAD^+^ cofactor specificity for wild-type *Ps*PTDH. (A) The K76 side chain cation (violet) has moved into position to form a bridging ion-pair to the α–and β–phosphates of the NAD^+^. (B) The flexible A74-D82 loop (red ribbon) has moved to form a cavity for the nicotinamide ring of NAD^+^. The enzyme-bound sulfite dianion is shown with the sulfur colored black.

#### The Role of K76 in Enzyme Activation

The K76 side chain is conserved among “known and putative *Ps*PTDHs in protein databases.” ^57,58^ The K76A substitution at wild-type *Ps*PTDH from *P. stutzeri* results in a decrease in *k*_cat_/*K*_NAD_ *K*_HPi_ for hydride transfer from 1.1 × 10^9^ M^–2^ s^–1^ for wild-type *Ps*PTDH to 2.9 × 10^6^ M^–2^ s^–1^ for the K76A variant.^39,58^ This corresponds to a 3.5 kcal/mol side-chain stabilization of the transition state for the reaction catalyzed by wild-type *Ps*PTDH. The K76 side chain interacts with enzyme-bound NAD^+^ but the K76A substitution results in only a small increase in *K*_NAD_ that corresponds to a 0.9 kcal/mol destabilization of the cofactor Michaelis complex. The main effect of this substitution is on *k*_cat_/*K*_HPi_ and corresponds to a 2.6 kcal/mol destabilization of the transition state for hydride transfer from HPO_3_^2–^ to enzyme-bound NAD^+^ at a site that is distant from K76 side chain (Figures 8 and 9). We conclude that the K76 side chain functions to stabilize the protein cavity that has evolved to provide optimal stabilization of the transition state for hydride transfer from HPO_3_^2–^ to NAD^+^ and that relaxation of the protein structure at the K76A variant results in a significant increase in the barrier for *Ps*PTDH-catalyzed hydride transfer.

These results show that the catalytic activity of *Ps*PTDH is reduced by a side chain substitution that destabilizes the closed enzyme **E**_C_ relative to the open form **E**_**O**_ (Scheme 7). Similar results have been reported for studies on the decarboxylation reaction catalyzed by yeast OMPDC, where substitutions of side chains at the phosphodianion gripper loop, which lock the substrate OMP into a protein cage, result in up to a 10^8^-fold decrease in *k*_cat_/*K*_m_ for decarboxylation at the distant pyrimidine ring of OMP.^50,59^ Studies on the isomerization reaction catalyzed by TIM have likewise shown that substitutions of side chains from loop 7 that stabilize the protein–substrate cage cause significant decreases in the kinetic parameters for enzyme-catalyzed isomerization of triosephosphates.^60–62^

## Conclusions

The results from studies on cofactor fragment activation of 17X-*Ps*PTDH-, *Cb*FDH- and *hl*GPDH-catalyzed hydride transfer to the truncated NR cofactor show that different regions of the whole cofactor activate these enzymes for catalysis of hydride transfer. This provides strong evidence that the total intrinsic binding energy for the NAD^+^ cofactor is substantially larger than that required to obtain the rate acceleration for enzyme-catalyzed hydride transfer to NAD^+^. We expect that studies on other dehydrogenases will show additional differences in the activating cofactor-driven conformational changes that make use of interactions with different regions of the NAD^+^ cofactor.

The results reported here, and in studies on many other enzymatic reactions over the past 20 years, demonstrate the existence of two types of protein interactions for enzymes that are activated for catalysis by interactions with nonreacting substrate fragments:^4–6^ (i) Binding interactions with the substrate fragment that function solely to stabilize the substrate Michaelis complex. (ii) Binding interactions with the substrate fragment that function to activate the protein for catalysis through stabilization of a catalytically active closed form, and which drive an activating protein conformational change (Scheme 7).^4–6^ Protein engineers have so-far worked to model only the former interactions but the later makes large contributions to the rate accelerations observed for Nature’s most proficient enzyme catalysts.

## Supplementary Material



## Notes

Figure S1: QTOF mass spectra for 17X-*Ps*PTDH from *P. stutzeri*; Figure S2: Michaelis–Menten plot for 17X-*Ps*PTDH-catalyzed hydride transfer reactions of 1.0 mM NAD^+^ at increasing [HPO_3_^2–^]; Figure S3: Michaelis–Menten plot for 17X-*Ps*PTDH-catalyzed hydride transfer reactions of 20 mM HPO_3_^2–^ at increasing [NAD^+^]; Figure S4: Effect of increasing concentrations of substrate NR and ADP activator on *v*/[E] for 17X-*Ps*PTDH-catalyzed hydride transfer reactions of 8 mM HPO_3_^2–^; Figure S5: Effect of increasing concentrations of substrate NR and ADP activator on *v*/[E] for 17X-*Ps*PTDH-catalyzed hydride transfer reactions of 16 mM HPO_3_^2–^ (PDF)

## Abbreviations

ADP: adenosine 5′-diphosphate
AMP: adenosine 5′-monophosphate
BSA: bovine serum albumin
GPDH: glycerol 3-phosphate dehydrogenase
G3P: glycerol 3-phosphate
DHAP: dihydroxyacetone phosphate
EtG: ethylene glycol
FDH: formate dehydrogenase
NADH: nicotinamide adenine dinucleotide (reduced form)
NAD^+^: nicotinamide adenine dinucleotide, (oxidized form)
NMNH: nicotinamide mononucleotide (reduced form)
NMN: nicotinamide mononucleotide (oxidized form)
NRH: nicotinamide riboside (reduced form)
NR: nicotinamide riboside (oxidized form)
OMPDC: orotidine 5′-monophosphate decarboxylase
TEA: triethanolamine
TIM: triosephosphate isomerase.
