# Supplementary material for: Enzyme Architecture: Activation of Phosphite Dehydrogenase-Catalyzed Hydride Transfer by NAD+ Cofactor Fragments
Source: Biochemistry. 2025 Nov 11;64(23):4581–93. doi: 10.1021/acs.biochem.5c00561 (PMC12676663; doi:10.1021/acs.biochem.5c00561)
Supplement: Supplementary file 1 [file bi5c00561_si_001.pdf]

SUPPORTING INFORMATION

Enzyme Architecture: Activation of Phosphite Dehydrogenase-Catalyzed Hydride Transfer by NAD$^+$ Cofactor Fragments.

Rania Hegazy[†] *and* John P. Richard[†,*]

[†]*Department of Chemistry, University at Buffalo, SUNY, Buffalo, New York 14260-3000 USA*

[*] *Author to whom correspondence should be addressed: EMAIL: jrichard@buffalo.edu*

Figure S1 shows mass spectra from LC/ESI-MS analysis performed on 17X-PTDH from *P. stutzeri* obtained using a QTOF LC/MS 6500 from Agilent. The protein was prepared for analysis by liquid chromatography over a Phenomenex Aeris C4 column (3.6 µm, 200 Å, 2.10 × 100 mm), using a 15 min linear gradient of 10–90% acetonitrile/water containing 0.1% HCOOH and a flow rate of 150 µL/min. The Figure on the left shows the charge ladder, and the Figure on the right shows the spectra obtained after deconvolution of the ladder using the Agilent BioConfirm software. The molecular weight determined for the protein (36764.1 Da) show good agreement with the calculated value (36764.4 Da) using the ProtParam tool,[1, 2] and assuming that the Gly-Ser-His tripeptide from the thrombin cleavage site remains attached to the N-terminal methionine.

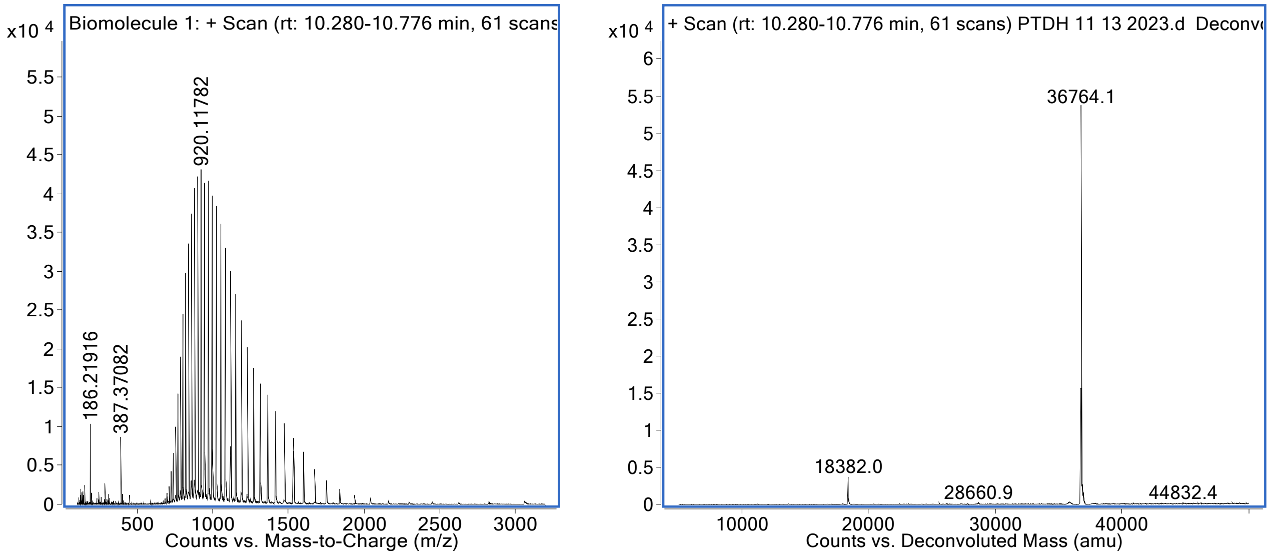

Figure S1. LC/ESI-MS analysis of 17X-PTDH from *P. stutzeri*. The charge ladders are shown on the spectrum on the left and the spectrum on the right was obtained after deconvolution of these ladders using the Agilent BioConfirm software. The calculated molecular weight is 36764.4 amu, and the measured molecular weight is 36764.1 amu.

Figure S2 shows the dependance of v/[E] on the concentration of phosphite dianion for PTDH-catalyzed reactions at a saturating concentration of 1.0 mM NAD^+ and Figure S3 shows the dependance of v/[E] on [NAD^+] for reactions at a constant nearly saturating concentration of 20 mM HPi. The kinetic parameters $k_{cat} = 2.3 \pm 0.02$ s^{-1} and $K_{HPi} = 4.1 \pm 0.1$ mM; and, $k_{cat} = 2.1 \pm 0.02$ s^{-1} and $K_{NAD} = 0.082 \pm 0.003$ mM were obtained, respectively from the fit of the kinetic data from Figures S2 and S3 to the Michaelis-Menten equation. Figures S4 and S5 show the increase in v/[E] with increasing concentrations of ADP activator for *Ps*PTDH-catalyzed hydride transfer reactions of 8 mM and 16 mM phosphite dianion, respectively, for reactions at different fixed concentrations of NR.

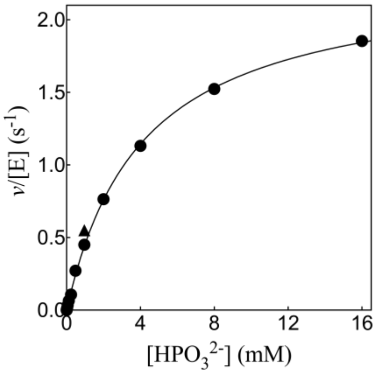

Figure S2. The increase in v_E/[E] with increasing concentration of phosphite dianion for PTDH-catalyzed hydride transfer reactions of 1.0 mM NAD^+ at 25 °C and pH 7.5 (36 mM TEA) and I = 0.30 (NaCl). The triangle shows data for reaction at 2.0 mM NAD^+.

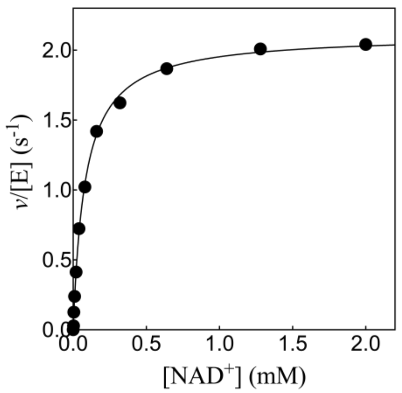

Figure S3. The increase in v_E/[E] with increasing concentration of NAD^+ for PTDH-catalyzed hydride transfer reactions of 20 mM phosphite dianion at 25 °C and pH 7.5 (36 mM TEA) and I = 0.3 (NaCl).

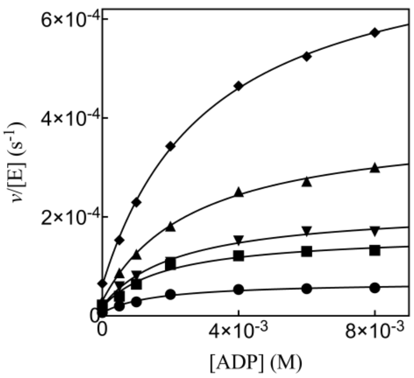

Figure S4. The effect of increasing [ADP] on *v*/[E] for *Ps*PTDH-catalyzed hydride transfer from 8 mM phosphite dianion to different fixed concentrations of NR. Key: 0.001 M NR, circles; 0.002 M NR, squares; 0.003 M NR, inverted triangles; 0.005 M NR, triangles; 0.01 M NR, diamonds.

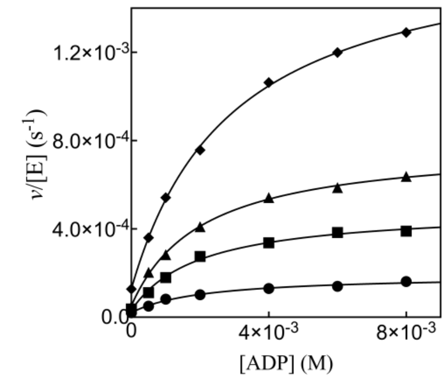

Figure S5. The effect of increasing [ADP] on *v*/[E] for *Ps*PTDH-catalyzed hydride transfer from 16 mM phosphite dianion to different fixed concentrations of NR. Key: 0.001 M NR, circles; 0.003 M NR, squares; 0.005 M NR, triangles; 0.01 M NR, diamonds.

REFERENCES

(1) Gasteiger, E., Hoogland, C., Gattiker, A., Duvaud, A., Wilkins, M. R., Appel, R. D., and Bairoch, A. Protein Identification and Analysis Tools on the ExPASy Server. *Proteomics Protocols Handbook* **2005**, 571-607

(2) Gasteiger, E., Gattiker, A., Hoogland, C., Ivanyi, I., Appel, R. D., and Bairoch, A. ExPASy: The proteomics server for in-depth protein knowledge and analysis. *Nuc. Acids Res* **2003**, *31*, 3784-3788
